# COVID-19 Vaccination Status Among Healthcare Workers and Its Effect on Disease Manifestations: A Study From Northeast India

**DOI:** 10.7759/cureus.25159

**Published:** 2022-05-20

**Authors:** Md Jamil, Prasanta K Bhattacharya, Bhupen Barman, K G Lynrah, Monaliza Lyngdoh, Iadarilang Tiewsoh, Annu Gupta, Ayan Mandal, Debashis P Sahoo, Varsha Sathees

**Affiliations:** 1 General Medicine, North Eastern Indira Gandhi Regional Institute of Health and Medical Sciences, Shillong, IND

**Keywords:** north east india, clinical manifestation, health-care worker, covid-19 vaccine, sars-cov-2

## Abstract

Background and objective

Since being declared a global pandemic, coronavirus disease 2019 (COVID-19) has led to millions of cases and deaths worldwide. Although severe acute respiratory syndrome coronavirus 2 (SARS-CoV-2) continues to wreak havoc on individuals, healthcare systems, and economies, the intensive vaccination strategies adopted by several countries have significantly slowed the progress and the severity of the disease. In this study, we aimed to determine the COVID-19 vaccination status among healthcare workers (HCWs) and examine the effects of vaccination on disease manifestations.

Materials and methods

This cross-sectional study was conducted at a teaching hospital in Northeast India from April 2021 to September 2021, during the second phase of the COVID-19 pandemic. HCWs employed in the hospital who were laboratory-confirmed cases of COVID-19 based on semiquantitative real-time reverse transcriptase-polymerase chain reaction (RT-PCR) or cartridge-based nucleic acid amplification test (CBNAAT) on oropharyngeal samples were included in the study. Data analysis was performed using Microsoft Excel (Microsoft Office Professional Plus 2019, Microsoft Corp., Redmond, WA)

Results

A total of 178 HCWs reported positive for COVID-19 infection during the study period. Of these, 42 (23.59%) were males and 136 were females (76.40%). Among them, 86 (48.32%) HCWs were fully vaccinated, 58 (32.58%) were partially vaccinated, and 34 (19.10%) were not vaccinated. Most of the HCWs experienced mild disease (145, 81.46%), and only four (2.24%) reported moderate to severe disease. Compared with unvaccinated HCWs, individuals who have had either one or two doses of vaccines were less likely to have moderate to severe disease or seek treatment at the hospital. On symptoms analysis, shortness of breath was found to be more common in unvaccinated individuals than in vaccinated patients, and anosmia and loss of taste were more common in vaccinated than in unvaccinated individuals. No deaths were reported among the participants included in this study.

Conclusions

Following the first and second waves of the COVID-19 pandemic, a substantial proportion of HCWs were infected with SARS-CoV-2, likely as a result of the acquisition of the virus in the community during the early phase of local spread. Fully vaccinated individuals with COVID-19 were more likely to be completely asymptomatic or only mildly symptomatic compared to unvaccinated HCWs.

## Introduction

Coronavirus diseases 2019 (COVID-19) is caused by a novel coronavirus called severe acute respiratory syndrome coronavirus 2 (SARS-CoV-2) [1]. It was first reported in Wuhan, Hubei Province, China in December 2019 [2]. Since the onset of the COVID-19 pandemic, more than 238 million people have been infected, leading to more than 4.8 million mortalities, as of October 9, 2021 [3]. In the absence of any specific treatment against the COVID-19 virus, vaccination remains the only viable option to combat this pandemic for now. The United States Food and Drug Administration (USFDA) gave its first approval for a vaccine against the COVID-19 on December 11, 2020, on an emergency use authorization basis, for the COVID-19 mRNA vaccine (BNT162b2). As of October 9, 2021, more than 6.47 billion doses of various COVID-19 vaccines have been administered worldwide [4]. Due to the shortage of vaccines in the immediate aftermath of the initial rollout of vaccines, only those people at high risk of getting an infection or at risk of developing severe disease were vaccinated on a priority basis. Healthcare workers (HCWs) directly involved in the care of COVID-19 patients face a higher risk of getting infected in comparison to the general population [5]. Hence, they were the first group of people to be vaccinated against COVID-19. In India, vaccination against COVID-19 was started on January 16, 2021, and as of October 9, 2021, more than 946 million doses of vaccine have been administered [6]. However, like in the case of any other vaccine, there has been vaccination hesitancy among the HCWs regarding the COVID-19 vaccine as well and, as a result, there have been many cases where HCWs diagnosed with COVID-19 were found to be unvaccinated [7].

Individuals diagnosed with COVID-19 may have protean manifestations and different clinical needs [8]. There have been scarce data from Northeast India regarding the COVID-19 pandemic [9-11]. Analysis of symptom profiles among individual COVID-19 patients following vaccination is valuable in terms of clinical utility, assessment and identification of risk groups (e.g., long COVID) for intervention, and the appropriate use of testing guidelines [12]. Against this background, the present study was conducted during the second wave of COVID-19 in India, which was mostly attributed due to the emergence of the Delta variant of the COVID-19 virus [13]. Our objectives were as follows: (1) to determine the COVID-19 vaccination status among HCWs at the time of COVID-19 diagnosis, and (2) to study the effect of the COVID-19 vaccination on disease manifestations.

## Materials and methods

The study was conducted at a tertiary care medical teaching institute in the state of Meghalaya in Northeast India. The study included cases diagnosed during the period from April 2021 to September 2021, which coincided with the second wave of the COVID-19 in the state of Meghalaya. Only those HCWs who are working in the institute where the study was conducted were included, and they were followed up for at least three weeks from the date of diagnosis. The study included 178 laboratory-confirmed cases of COVID-19 based on either semiquantitative real-time reverse transcriptase-polymerase chain reaction (RT-PCR) or cartridge-based nucleic acid amplification test (CBNAAT) on oropharyngeal samples. All patients were under the direct supervision of the treating institute. Patients who were treated outside the institute were excluded from the study. For the purpose of comparison, the cases were classified into three groups based on the vaccination status:

Category-A: Nonvaccinated - Patients who were either not vaccinated or received their first dose of vaccine within seven days of the diagnosis of COVID-19.

Category-B: Partially vaccinated - Patients who either received the first dose of vaccine eight or more days prior to the COVID-19 diagnosis or received the second dose of vaccine within seven days of the diagnosis of COVID-19.

Category-C: Fully vaccinated - Patients who received the second dose of vaccine eight or more days prior to the diagnosis of COVID-19.

Data related to demographic details, vaccination status, clinical manifestations, and disease outcomes were collected. Ethical approval was obtained from the Institution Ethics Committee, North Eastern Indira Gandhi Regional Institute of Health and Medical Sciences vide letter No. NEIGR/IEC/M15/F20/2021 dated August 28, 2021, and informed written consent was obtained from all study participants.

## Results

A total of 178 cases were included in the present study. Of them, 42 (23.59%) were males and 136 (76.40%) were females, with a male-to-female ratio of 0.31:1. Most of the study patients were nursing officers (n=102, 57.30%) followed by resident doctors (n=37, 16.29%), technicians (n=17, 7.86%), housekeeping staff (n=12, 6.74%), and faculty members (n=10, 5.62%). The number of cases in Category-A, Category-B, and Category-C was 58 (32.58%), 34 (19.10%), and 86 (48.32%) respectively. All those who were vaccinated had received only Covishield [ChAdOx1 nCoV-19 Corona Virus Vaccine (Recombinant)] manufactured by the Serum Institute of India Pvt Ltd. The vaccination status among the different categories of the staff at the time of COVID-19 diagnosis is shown in Table 1. Characteristics such as the mean age, gender distribution, and severity of disease in the different categories are shown in Table 2.

**Table 1:** Vaccination status among various HCW categories at the time of diagnosis of COVID-19 COVID-19: coronavirus disease 2019; HCW: healthcare worker

Categories of HCW	Vaccination status at the time of COVID-19 diagnosis			
	No vaccination, n (%)	Incomplete vaccination, n (%)	Complete vaccination, n (%)	Total, n (%)
Nursing staff	35 (19.66%)	28 (15.73%)	39 (21.91%)	102 (57.30%)
Resident doctors	4 (2.25%)	4 (2.25%)	29 (16.29%)	37 (20.79%)
Faculty members	0	0	10 (5.61%)	10 (5.62%)
Housekeeping staff	10 (5.61%)	1 (0.56%)	1 (0.56%)	12 (6.74%)
Technicians and other supporting staff	9 (5.06%)	1 (0.56%)	7 (3.93%)	17 (9.55%)
Total	58 (32.59%)	34 (19.10%)	86 (48.31%)	178 (100%)

**Table 2:** Patient profiles in different categories as per vaccination status HCW: healthcare worker; SD: standard deviation

Patient profiles		Category of HCWs as per vaccination status		
		Category-A	Category-B	Category-C
Number of patients		58	34	86
Male-to-female ratio		0.23:1	0.06:1	0.51:1
Age, years, mean ±SD		33.47 ±5.60	36.47 ±8.21	35.19 ±7.92
Severity of the disease (number of cases)	Asymptomatic	10	3	16
	Mild	45	30	70
	Moderate	2	1	0
	Severe	1	0	0
	Total	58 (32.59%)	34 (19.10%)	86 (48.31%)

**Figure 1:**
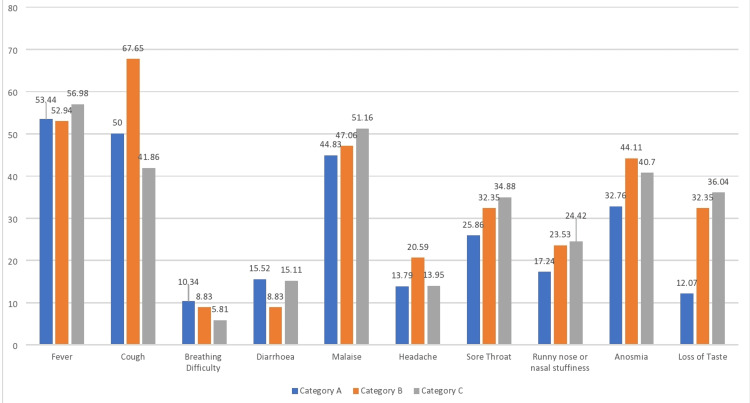
Symptoms in COVID-19-infected unvaccinated (Category-A), partially vaccinated (Category-B), and vaccinated (Category-C) patients COVID-19: coronavirus disease 2019

Figure 1 illustrates symptoms in various categories of HCWs who were diagnosed with COVID-19.

## Discussion

In the absence of an effective and sustainable infection control strategy and the non-availability of a specific treatment against the COVID-19, effective vaccination remains the only viable option to fight against the COVID-19 pandemic. The sense of urgency to have an effective vaccine against COVID-19 coupled with great human effort has led to the development of multiple vaccines against COVID-19 within a year of the first reported case of the ongoing COVID-19 pandemic. As of February 2022, India has authorized three vaccines against SARS-CoV-2: Covishield (AstraZeneca's vaccine manufactured by the Serum Institute of India), Covaxin (manufactured by Bharat Biotech Limited), and Sputnik V [14]. But coronaviruses are known to undergo genetic mutation as they propagate, and it has happened in the case of SARS-CoV-2 as well, resulting in the appearance of multiple variants of the virus leading to multiple waves of increased cases and reinfections [15-18]. The appearance of multiple variants also has the potential to render the existing vaccines ineffective [19].

Even though vaccines against COVID-19 were made available within the shortest possible period, many people including HCWs remained hesitant to get vaccinated due to doubts regarding the efficacy and safety of the available vaccines. In the present study, 32.59% of HCWs had not received any dose of vaccine at the time of COVID-19 diagnosis, 19.10% were partially vaccinated, and only 48.31% were completely vaccinated. Among the categories of HCWs, the housekeeping staff was the most unvaccinated group followed by the nursing staff. Doctors including faculty members and residents were predominantly vaccinated at the time of COVID-19 diagnosis. Vaccine hesitancy was found to be higher among the nursing staff and housekeeping staff in the present study, which is similar to the findings reported in other studies [20,21].

All HCWs in the fully vaccinated category had either mild disease or were asymptomatic. Among the partially vaccinated or completely unvaccinated, 4.34% of cases developed moderate to severe disease. No mortality was reported in the present study in any of the categories. Similar findings were reported by other studies where most of the HCWs with breakthrough infections after receiving the Oxford-Astra Zeneca vaccine were either asymptomatic or had mild disease [22,23].

In a study by Teran et al. involving 75 skilled nursing care facilities in Chicago, among 627 persons with SARS-CoV-2 infection since vaccination began, 22 (4%) were identified as residents and staff members of skilled nursing facilities. On further analysis, nearly two-thirds (14/22, 64%) of the patients were found asymptomatic with two COVID-19-related hospitalizations and one death [24]. Similar results were also reported by different studies across India; however, none of these studies reported any deaths related to COVID-19 among HCWs who received two doses of the vaccine (Table 3) [25-28]. The possible hypothesis for this post-vaccination COVID-19 infection could be ascribed to the emergence of new COVID-19 variants, which may bypass vaccine-induced immunity [29]. It is reassuring that the majority of infections seen in our facility were either asymptomatic or mild.

**Table 3:** Comparison of disease manifestations among HCWs after two doses of COVID-19 vaccine as reported by different studies COVID-19: coronavirus disease 2019; HCW: healthcare worker

Variables	Mohith et al. [25]	Vaishya et al. [26]	Vaishya et al. [27]	Tyagi et al. [28]	Present study
Type of the vaccine	ChAdOx1 nCoV-19 (Covishield)	ChAdOx1 nCoV-19 (Covishield)	Covishield and Covaxin	Covishield and Covaxin	ChAdOx1 nCoV-19 (Covishield)
Number of HCWs enrolled	36	85	28,342	123	178
Percentage of asymptomatic or mild cases	88.9%	100%	_	94.7%	100%
Rate of hospitalization	11%	0.06%	0.28%	0.93%	0%
Oxygen requirement	Nil	Nil	-	0.93%	0%
Severe disease requiring oxygen supplementation or ICU admission	Nil	Nil	0.01%	0.93%	0%
Mortality	Nil	Nil	Nil	-	Nil

## Conclusions

Based on our findings, COVID-19 vaccination acceptance is not uniform among the different categories of the HCWs. Vaccination acceptance is almost universal among doctors but less among the nursing and housekeeping staff. Those who were completely vaccinated were found to have negligible levels of serious disease when compared to those who were either unvaccinated or incompletely vaccinated. These findings suggest that widespread and effective vaccination among HCWs provides a safe environment, even in the setting of a high rate of SARS-CoV-2 infection in the community.
